# Psychological Impact of COVID-19 Confinement and Its Relationship with Meditation

**DOI:** 10.3390/ijerph17186642

**Published:** 2020-09-11

**Authors:** Óliver Jiménez, Laura C. Sánchez-Sánchez, José M. García-Montes

**Affiliations:** 1 Faculty of Psychology and Speech Therapy, Campus de Teatinos, University of Málaga, 29071 Málaga, Spain; oliverjjimenez@uma.es; 2 Departament of Evolutionary and Educational Psychology, Faculty of Science Education and Sport, University of Granada, Calle Santander, N° 1, 52071 Melilla, Spain; 3 Departament of Psychology, University of Almeria, Carretera Sacramento, S/N, La Cañada de San Urbano, 04120 Almería, Spain; jgmontes@ual.es

**Keywords:** coronavirus, COVID-19, stress, anxiety, depression, mindfulness, mental health, Spain, psychological impact, confinement

## Abstract

The objective of this study was to evaluate the psychological impact of confinement due to the COVID-19 pandemic, considering any protective factors, such as the practice of meditation or self-compassion, and their relationship with different lifestyles and circumstances of adults residing in Spain. A cross-sectional study was done using an anonymous online survey in which 412 participants filled out the Depression, Anxiety and Stress Scale-2; the Impact of Events Scale; and the Self-Compassion Scale-Short Form, reporting severe symptomatology of posttraumatic stress and mild anxiety and depression. Quality of cohabitation and age were found to be key variables in the psychological impact of confinement. The impact of confinement was more negative for those who reported very poor cohabitation as opposed to very good (*F* (3, 405) = 30.75, *p* ≤ 0.001, *d* = 2.44, *r* = 0.054) or for those under 35 years of age compared to those over 46 (*F* (2, 409) = 5.14, *p* = 0.006, *d* = 0.36). Practicing meditation was not revealed as a protective factor, but self-compassion was related to better cohabitation during confinement (*F* (3, 403) = 11.83, *p* ≤ 0.001, *d* = 1.05). These results could be relevant in designing psychological interventions to improve coping and mental health in other situations similar to confinement.

## 1. Introduction

In December 2019, an outbreak of pneumonia with an unknown etiology was announced in Wuhan (China), soon becoming a global pandemic and surpassing the severe acute respiratory syndrome (SARS) of 2003 [1]. The World Health Organization (WHO) identified this new pneumonia as a new coronavirus called SARS-COV-2 or as it is commonly called, COVID-19. Since the first cases were recorded in December 2019, the number has increased exponentially throughout the world and a public health emergency of international interest was declared on 30 January 2020 [2]. By March 11th, COVID-19 had already been declared a worldwide pandemic with 118,000 cases in 114 countries, although 90% of them were concentrated in China and the Republic of Korea [3]. At the time of writing, 28 June 2020, global coronavirus cases surpassed 10 million, with over half a million deaths. In Europe, over 2.5 million people have been infected, with almost 200,000 deaths, and Spain has had the third most cases in Europe, with 248,770, and the sixth most deaths, with 28,343 [4].

This rapid expansion of the virus is partly due to its high transmissivity, which has an estimated basic reproduction number of 2.24 to 3.58, meaning that an individual who is a transmitter of the virus could infect from two to four others [5], even though they do not show any symptomatology [6], and its incubation period varies from two to 14 days, with an average of seven days [7]. The symptomatology of those infected is fever, dry cough, sore throat, difficulties breathing, fatigue, nausea, vomiting, and diarrhea. The most commonly reported comorbidities are hypertension, diabetes, headache, dizziness, fatigue, myalgia, hypo/anosmia, and gustatory dysfunction [8]. Regarding psychiatric and neuropsychiatric presentations, it seems that in its acute stage, SARS-CoV-2 might cause delirium in a significant proportion of patients. It might also cause depression, anxiety, fatigue, posttraumatic stress disorder, and more rarely, in the long-term, neuropsychiatric syndromes [9].

COVID-19 can infect people of all ages, but men over 70 with preexisting conditions, such as asthma, diabetes, or heart disease, are the most susceptible, reporting an >8% mortality rate [5]. Women are less susceptible to infection and because of their distinctive immune system, severity and mortality are much lower in children than in adults [5].

In view of all of the above and especially because of the rapid expansion of the epidemic, many countries have decreed a state of emergency with mass quarantine of their citizens. In the particular case of Spain, this state of emergency has imposed a closure of establishments, ceasing of all except essential activity, and home confinement of the population during a period of time depending mainly on the evolution of the number of people infected and the expansion of the virus. In this respect, recent studies [10,11] have mentioned that understanding the effect this quarantine has on the population is of great importance, especially in countries as severely affected as Spain. Quarantine could be understood as an important source of stress as it is unpredictable and uncontrollable, which are core features of the psychobiological construct of stress [12].

A recent review [10] on the psychological impact of quarantining for SARS, H1N1 flue, and other causes reported fear in over 20% of the population, 18% sadness and 10% blame, and a high prevalence of distress, stress, depression, or bad mood. Some of the factors generating stress during the SARS quarantine were duration and fear of infection. Some studies showed a positive association between length of quarantine and increase in posttraumatic stress symptoms, avoidance behaviors, or anger [10]. In recent studies on the impact of COVID-19, women have manifested a greater psychological impact and worse mental health [13]. Young people and students are also more vulnerable because of schools and universities closing, as well as social isolation, altering the normal development of their education [14].

In such a situation, systematic psychological self-care should have a high priority in coping with the detrimental impacts of COVID-19 and social distancing. Thus, acknowledging one’s own limitations with compassion as a part of the shared human experience could have a positive impact on mental health. In this respect, possible protective factors could be mindfulness or self-compassion, since, as mentioned in a recent review [15], it seems that mindfulness-based treatments are effective in reducing posttraumatic stress disorder (PTSD) symptoms and the Mindfulness-based Stress Reduction Program was the one that provided the most evidence [16]. In this vein, another review [17] emphasized the promising results associating an increase in self-compassion with reduction in PTSD symptoms, reducing the impact of exposure to trauma.

Therefore, and since to date there are few studies reporting on the emotional impact the pandemic is having on the psychological health of those confined, the objective of this study was to explore the impact of confinement due to COVID-19 on the mental and emotional health of adult Spanish-speaking residents of Spain. In particular, we intended to inquire into the possible impact of confinement on anxiety, depression, and posttraumatic stress and whether practice of some type of meditation or self-compassion could be a protective factor against this impact. Then, any between-group differences in these variables would be analyzed by sample sociodemographic variables (age, cohabitation, etc.) and finally, the relationships between the abovementioned variables (anxiety, depression, stress, self-compassion) and the sociodemographic variables selected (e.g., age or treatment of those confined) would be explored.

## 2. Materials and Methods

### 2.1. Participants

A total of 412 adults from 63 Spanish provinces, with a mean age of 40.48 (*SD* = 10.79) participated in the survey out of a total of 420 (finalization rate: 98.09%). Most of the participants were Spanish (91%), women (84.5%), aged 18 to 73, with no changes in their employment because of COVID-19 (73.8%), a university education (42%), and did not practice any type of meditation (69.9%). The rest of the sociodemographic variables recorded are presented in Table 1.

### 2.2. Instruments

Data collected for the study were sociodemographic (gender, age, nationality, marital status, occupation, employment, education); health (whether under treatment or not and if so, what); quality of cohabitation during the pandemic; cohabitation with a case of COVID-19; whether religious or not and if so, practicing or not; and whether they practiced mediation and if so, what type.

Mental health was evaluated using the following scales:

Depression, Anxiety and Stress Scale-21 (DASS-21). The DASS-21 consists of 21 items evaluating the frequency and severity of emotional symptoms experienced during the previous week, using a self-report scale with four choices from 0 (Never) to 3 (Almost always). It is made up of three subscales, Depression, Anxiety, and Stress, with seven items each. A high score on each subscale means stronger symptomatology ([18,19] Spanish adaptation). Internal consistency of the Spanish adaptation has an α = 0.93 for the Depression scale, α = 0.86 for Anxiety, α = 0.91 for Stress, and α = 0.96 for the total scale [19].

Impact of Events Scale (IES). The IES consists of 15 items evaluating subjective stress from stressful and or traumatic experiences in the previous week, with two subscales, Intrusion and Avoidance, with seven and eight items, respectively ([20,21] Spanish adaptation). It is a four-choice self-report scale varying from 0 (never) to 4 (often). Based on the sum of all the items, the cutoff points are 8.6 to 19 for mild symptoms and 19 or over for severe symptoms. The internal consistency of the Spanish adaptation has an α =.86 for the total scale, α = 0.76 for Intrusion and α = 0.82 for Avoidance [21].

Self-Compassion Scale-Short Form (SCS-SF). The short form of the scale is made up of 12 items that evaluate self-compassion ([22,23] Spanish adaptation). It consists of three main subscales (Mindfulness, Self-Kindness, and Common Humanity) and their opposites (Over-identification, Self-Judgment, and Isolation). The scale has five answer choices from 1 (Almost never) to 5 (Almost always). A higher score is interpreted as more self-compassion. The internal consistency of the Spanish adaptation is α = 0.71 for Self-Judgment, α = 0.75 for Common Humanity, α = 0.77 for Isolation, α = 0.74 for Mindfulness, α = 0.76 for Over-Identification, α = 0.73 for Self-Kindness, and α = 0.85 for the total scale [23].

### 2.3. Procedure

A cross-sectional study was conducted from 4 April 2020 to 1 May 2020. The sample was recruited by non-probabilistic snowball sampling, since it was an exploratory study in which rapid access to data was needed. First, contacts of the research team were invited to participate and then, these contacts were asked to forward the invitation to their contact networks. An online survey form was prepared using Google Forms, which included information on the study; telling the participants that they would not receive any compensation for taking part in it; a series of sociodemographic and additional variables of interest; and the DASS-21, IES, and SCS scales. The study protocol was approved by the University of Almería Bioethics Committee (No. UALBIO2020/032) and the participants signed their consent after being informed of the implications of their participation and the confidentiality of the data provided, according to the Helsinki Declaration and the Organic Law on Data Protection 15/1999 of December 13th.

### 2.4. Data Analysis

All the analyses were performed with SPSS version 26.0 (IBM Corp., Armonk, NY, USA) and the descriptive results of the quantitative variables were reported with mean and standard deviation. An ANOVA was done to evaluate the between-group differences in the sociodemographic variables and then, a Student’s *t*-test was applied for comparisons between each pair of groups. Further, the effect size was calculated for those that were statistically significant. Finally, the bivariate correlation was calculated to evaluate the relationship of the different sociodemographic ordinal variables and subscales. In all the analyses, *p* < 0.05 was considered statistically significant.

## 3. Results

### 3.1. Impact of Confinement on Anxiety, Depression, Posttraumatic Stress, and Self-Compassion

The general mean on the DASS-21 scale for participant Stress was 6.67 (*SD* = 5.25), 3.88 (*SD* = 4.51) for Anxiety and 4.51 (*SD* = 4.54) for Depression, which reflects mild depression and anxiety, and the stress score was not symptomatic. On the IES scale, the mean for Intrusion was 12.61 (*S* = 7.39), 13.66 (*SD* = 7.62) for Avoidance, and on the total scale, it was 26.22 (*SD* = 13.78), which shows severe symptomatology. Finally, on the SCS scale, mean Self-Kindness was 6.46 (*S* = 1.67) and for Common Humanity, it was 6.44 (*SD* = 1.60) and in Mindfulness, it was 6.69 (*SD* = 1.69).

### 3.2. Between-Group Differences in Anxiety, Depression, Stress, and Self-Compassion by Sociodemographic Variables. Analysis of Each Questionnaire

An ANOVA was done with the most significant variables to test for differences between the sociodemographic variables and psychological scales. This section only shows the data for those sociodemographic variables that were statistically significant. To be able to compare the groups by age, a new variable was created a posteriori by grouping participants by age into three groups of equivalent size by the quartiles found from the cutoff points. This new variable, “Age Group”, was divided into Young Adults (18 to 35), Adults (36 to 45), and Middle Aged (Over 46). In addition, and since one of the objectives of this study was to test the importance of meditation on the psychological impact of confinement, this variable was also included for testing.

#### 3.2.1. DASS-21

After the ANOVA and meeting the assumptions of normality and homogeneity of variance, statistically significant differences were found between psychological variables and sociodemographic variables.

In Stress, significant differences were found between age groups [*F* (2, 409) = 5.14, *p* =0.006], particularly between Young Adults and Adults (*M* = 7.73, *SD* = 5.20 vs. *M* = 6.44, *SD* = 4.83, *d* = 0.25, *r* = 0.000) and between Young Adults and Middle Aged (*M* = 7.73, *SD* = 5.20 vs. *M* = 5.76, *SD* = 5.54, *d* = 0.36, *r* = 0.000). Young adults showed higher scores of Stress, with a small effect size in both comparisons. Statistically significant differences were also found for Stress by cohabitation quality [*F* (3, 405) = 30.75, *p* ≤ 0.001], between Very Good and Good (*M* = 4.27, *SD* = 4.55 vs. *M* = 6.60, *SD* = 4.72, *d* = 0.49, *r* = 0.001), Very Good and Poor (*M* = 4.27, *SD* = 4.55 vs. *M* = 11.88, *SD* = 5.08, *d* = 1.61, *r* = 0.018), between Very Good and Very Poor (*M* = 4.27, *SD* = 4.55 vs. M = 15.75, *SD* = 7.93, *d* = 2.44, *r* = 0.054), between Good and Poor (*M* = 6.60, *SD* = 4.72 vs. *M* = 11.88, *SD* = 5.08, *d* = 1.10, *r* = 0.004), and between Good and Very Poor (*M* = 6.60, *SD* = 4.72 vs. 15.75, *SD* = 7.93, *d* = 1.91, *r* = 0.014). The worse cohabitation was, the higher the scores in Stress in all the between-group comparisons, with a small effect size between Very Good and Good and large for the rest.

No significant differences were found in Anxiety in any of the variables selected.

Statistically significant differences were found in Depression between age groups [*F* (2, 409) = 3.41, *p* = 0.034] between Young Adults and Middle Aged (*M* = 5.21, *SD* = 4.64 vs. *M* = 3.79, *SD* = 4.47, *d* = 0.31, *r* = 0.000). Again, Young Adults scored higher on the Depression scale than Middle Aged, but with a small effect size.

#### 3.2.2. IES

Statistically significant between-group differences were observed in the Intrusion scale by education level [*F* (5, 399) = 3.19, *p* = 0.008]—in particular, between the group with Primary and Postgraduate Education (M = 17.22, DT = 6.55 vs. M = 10.88, DT = 6.98, *d* = 0.91, *r* = 0.007), between Secondary Education and Postgraduate (*M* = 17.22, *SD* = 6.55 vs. *M* = 10.88, *SD* = 6.98, *d* = 0.91, *r* = 0.007), between Secondary and High School (*M* = 16.18, *SD* = 7.81 vs. *M* = 12.10, *SD* = 6.91, *d* = 0.55, *r* = 0.006), and between Secondary and Postgraduate (*M* = 16.18, *SD* = 7.81 vs. *M* = 10.88, *SD* = 6.98, *d* = 0.74, *r* = 0.004). In all the comparisons with significant differences, the score on Intrusion was higher in the groups with lower education, with a moderate effect size, and large between Primary and Postgraduate. Statistically significant between-group differences were found in type of treatment [*F* (3, 401) = 5.66, *p* = 0.001] between those who had None and those who had one Medical (*M* = 11.73, *SD* = 7.08 vs. *M* = 14.89, *SD* = 7.84, *d* = 0.43, *r* = 0.001) and between those who had None and those who had one Psychological (*M* = 11.73, *SD* = 7.08 vs. *M* = 15.35, *SD* = 8.02, *d* = 0.50, *r* = 0.001). In both cases, the score on the Intrusion scale was higher in those participants who were under some type of treatment than those who were not, with a small and moderate effect size, respectively. The analysis by cohabitation quality showed significant between-group differences [*F* (3, 398) = 13.59, *p* ≤ 0.001], specifically between Very Good and Good (*M* = 9.67, *SD* = 7.33 vs. *M* = 12.84, *SD* = 6.99, *d* = 0.44, *r* = 0.001), between Very Good and Poor (*M* = 9.67, *SD* = 7.33 vs. *M* = 17.57, *SD* = 6.91, *d* = 1.09, *r* = 0.008), between Very Good and Very Poor (*M* = 9.67, *SD* = 7.33 vs. *M* = 18.25, *SD* = 7.88, *d* = 1.16, *r* = 0.013), and between Good and Poor (*M* = 12.84, *SD* = 6.99 vs. *M* = 17.57, *SD* = 6.91, *d* = 0.67, *r* = 0.002). In all the comparisons, the score on the Intrusion scale was higher in the groups that reported worse cohabitation, with a small effect size between Very Good and Good, large between Very Good and Poor and Very Good and Very Poor, and moderate between Good and Poor. Statistically significant between-group differences were also found by occupation [*F* (6, 398) = 2.91, *p*< 0.05] between Student and Employee (*M* = 9.03, *SD* = 6.27 vs. *M* = 12.19, *SD* = 7.26, *d* = 0.44, *r* = 0.001), Student and Government Employee (*M* = 9.03, *SD* = 6.27 vs. *M* = 12.86, *SD* = 7.58, *d* = 0.53, *r* = 0.003), Student and Unemployed (*M* = 9.03, *SD* = 6.27 vs. *M* = 15.11, *SD* = 7.28, *d* = 0.88, *r* = 0.010), Student and Retired (*M* = 9.03, *SD* = 6.27 vs. *M* = 16.05, *SD* = 8.77, *d* = 0.97, *r* = 0.019), Unemployed and Employee (*M* = 15.11, *SD* = 7.28 vs. M = 12.19, *SD* = 7.26, *d* = 0.40, *r* = 0.001), and Retired and Employee (*M* = 16.05, *SD* = 8.77 vs. *M* = 12.19, *SD* = 7.26, *d* = 0.52, *r* = 0.001). In general, the results show that Students scored lower in Intrusion than the rest of the occupations, with those who were Retired scoring highest, with a small effect size between Student and Employee and Unemployed and Employee, moderate between Student and Government employee and Retired and Employee, and large between Student and Unemployed and Student and Retired. Lastly, statistically significant differences [*F* (1, 400) = 8.73, *p* < 0.05] were found in those who had been in contact with someone with COVID-19 and those who had not (*M* = 15.28, *SD* = 7.56 vs. *M* = 12.16, *SD* = 7.30, *d* = 0.42, *r* = 0.000), with those who had been in contact with someone COVID-19 positive showing more Intrusion with a small effect size.

On the Avoidance scale, statistically significant between-group differences were found by type of treatment [*F* (3, 398) = 4.15, *p* = 0.006], in particular, between None and Medical (*M* = 12.88, *SD* = 7.48 vs. *M* = 16.01, *SD* = 8.04, *d* = 0.41, *r* = 0.000) and between None and Psychological (*M* = 12.88, *SD* = 7.48 vs. *M* = 16.00, *SD* = 7.18, *d* = 0.41, *r* = 0.001). In both comparisons, participants who were under some type of treatment scored higher on the Avoidance scale than those who were not, with a small effect size. Statistically significant between-group differences were also observed in the analyses by quality of cohabitation [*F* (3, 395) = 8.26, *p* ≤ 0.001], specifically between Very Good and Good (*M* = 10.93, *SD* = 7.33 vs. *M* = 14.07, *SD* = 7.50, *d* = 0.42, *r* = 0.001), between Very Good and Poor (*M* = 10.93, *SD* = 7.33 vs. *M* = 17.21, *SD* = 7.29, *d* = 0.85, *r* = 0.005), and between Good and Poor (*M* = 14.07, *SD* = 7.50 vs. *M* = 17.21, *SD* = 7.29, *d* = 0.42, *r* = 0.001). In all of the comparisons, the score on the Avoidance scale was higher in those groups with worse cohabitation, showing a small effect size, except between Very Good and Poor, which was large. Finally, statistically significant differences [*F* (1, 397) = 6.48, *p* < 0.05] were found in those who had been in contact with someone with COVID-19 and those who had not (*M* = 16.05, *SD* = 7.40 vs. *M* = 13.25, *SD* = 7.61, *d* = 0.37, *r* = 0.000), where those who had been in contact with someone COVID-19 positive showed stronger Avoidance with a small effect size.

Finally, in the total IES score, statistically significant between-group differences were found with regard to cohabitation quality [*F* (3, 390) = 12.05, *p* ≤ 0.001], specifically between those who had Very Good and Good cohabitation (*M* = 20.60, *SD* = 13.26 vs. *M* = 26.93, *SD* = 13.39, *d* = 0.47, *r* = 0.001), between Very Good and Poor (*M* = 20.60, *SD* = 13.26 vs. *M* = 34.36, *SD* = 12.51, *d* = 1.05, *r* = 0.008) and between Very Good and Very Poor (*M* = 20.60, *SD* = 13.26 vs. *M* = 35.25, *SD* = 12.01, *d* = 1.10, *r* = 0.012), and between Good and Poor (*M* = 26.93, *SD* = 13.39 vs. *M* = 34.36, *SD* = 12.51, *d* = 0.55, *r* = 0.001). The results show that the Total IES scores were lower in the groups with the best cohabitation, with a large effect size, except between Very Good and Good, which was small, and between Good and Poor, which was moderate. Differences were found with regard to occupation [*F* (6, 390) = 2.43, *p* < 0.05] between Student and Unemployed (*M* = 20.54, *SD* = 12.96 vs. *M* = 30.75, *SD* = 13.53, *d* = 0.76, *r* = 0.008), Student and Retired (*M* = 20.54, *SD* = 12.96 vs. *M* = 31.47, *SD* = 15.44, *d* = 0.78, *r* = 0.013), Student and Other Occupation (*M* = 20.54, *SD* = 12.96 vs. *M* = 30.54, *SD* = 17.11, *d* = 0.70, *r* = 0.012), and Employee and Unemployed (*M* = 25.51, *SD* = 13.56 vs. *M* = 30.75, *SD* = 13.53, *d* = 0.38, *r* = 0.001). Students scored lower in Total IES, especially compared to Retired, who had a higher score on Total IES, with a moderate effect size, except between Employee and Unemployed, which was small. Finally, statistically significant differences [*F* (1, 392) = 8.63, *p* < 0.05] were found for those who had been in contact with someone with COVID-19 and those who had not been (*M* = 31.20, *SD* = 13.45 vs. *M* = 25.35, *SD* = 13.71, *d* = 0.42, *r* = 0.000), where those who had been in contact with someone COVID-19 positive had a higher Total IES, with a small effect size.

#### 3.2.3. SCS

In the first of the SCS subscales, Self-kindness, significant differences were found by level of education [*F* (5, 404) = 2.91, *p* = 0.013] between those who had a Secondary education and University (*M* = 5.61, *SD* = 1.63 vs. *M* = 6.48, *SD* = 1.52, *d* = 0.56, *r* = 0.002), between Secondary and Postgraduate (*M* = 5.61, *SD* = 1.63 vs. *M* = 6.84, *SD* = 1.89, *d* = 0.66, *r* = 0.003), and between Professional Training and Postgraduate (*M* = 6.23, *SD* = 1.68 vs. *M* = 6.84, *SD* = 1.89, *d* = 0.33, *r* = 0.001). As the results show, the higher the education, the higher the score on Self-kindness, with a moderate effect size, except between Professional Training and Postgraduate, which was small. The analysis by treatment received showed statistically significant between-group differences [*F* (3, 406) = 8.65, *p* ≤ 0.001] between None and Medical (*M* = 6.70, *SD* = 1.56 vs. *M* = 5.91, *SD* = 1.63, *d* = 0.49, *r* = 0.001) and between None and Psychological (*M* = 6.70, *SD* = 1.56 vs. *M* = 5.46, *SD* = 1.87, *d* = 0.77, *r* = 0.002). These results show that Self-kindness is greater in those who are not under treatment, with a small effect size between None and Medical and moderate between None and Psychological. With regard to the quality of cohabitation, significant differences [*F* (3, 403) = 11.83, *p* ≤ 0.001] were found between Very Good and Good cohabitation (*M* = 6.91, *SD* = 1.64 vs. *M* = 6.51, *SD* = 1.59, *d* = 0.24, *r* = 0.000), between Very Good and Poor (*M* = 6.91, *SD* = 1.64 vs. *M* = 5.18, *SD* = 1.58, *d* = 1.05, *r* = 0.008), and between Good and Poor (*M* = 6.51, *SD* = 1.59 vs. *M* = 5.18, *SD* = 1.58, *d* = 0.83, *r* = 0.002). As the results show, in the groups with better cohabitation, the scores in Self-kindness were also higher, with a small effect size between Very Good and Good and large in the rest of the comparisons. Insofar as occupation, significant differences [*F* (6, 403) = 2.17, *p* = 0.04] were found between Unemployed and Housewife/husband (*M* = 5.76, *SD* = 1.78 vs. M = 7.15, *SD* = 2.06, *d* = 0.75, *r* = 0.010), Unemployed and Student (*M* = 5.76, *SD* = 1.78 vs. M = 6.79, *SD* = 1.67, *d* = 0.58, *r* = 0.005), Unemployed and Employed (*M* = 5.76, *SD* = 1.78 vs. *M* = 6.51, *SD* = 1.60, *d* = 0.45, *r* = 0.001), and Unemployed and Government Employee (*M* = 5.76, *SD* = 1.78 vs. *M* = 6.61, *SD* = 1.67, *d* = 0.49, *r* = 0.002). Unemployed, as the results show, scored lower in Self-kindness compared to the rest of the occupations, showing a moderate effect size. Statistically significant differences were also observed by type of meditation [*F* (4, 397) = 5.31, *p* ≤ 0.001], in particular, between the None and Mindfulness groups (*M* = 6.28, *SD* = 1.55 vs. *M* = 7.09, *SD* = 1.92, *d* = 0.50, *r* = 0.001), between None and Transcendental (*M* = 6.28, *SD* = 1.55 vs. *M* = 7.62, *SD* = 2.18, *d* = 0.85, *r* = 0.002), between Mindfulness and Vipassana (*M* = 7.09, *SD* = 1.92 vs. *M* = 5.50, *SD* = 1.30, *d* = 0.84, *r* = 0.009), and between Vipassana and Transcendental (*M* = 5.50, *SD* = 1.30 vs. M = 7.62, *SD* = 2.18, *d* = 1.13, *r* = 0.088). In the groups that practiced meditation, the scores in Self-kindness were higher, with a moderate effect size between None and Mindfulness and large in the rest of the comparisons. Lastly, statistically significant differences [*F* (1, 405) = 9.54, *p* < 0.05] were found between persons who had been in contact with someone diagnosed with COVID-19 and those who had not (*M* = 6.35, *SD* = 1.65 vs. *M =* 6.46, *SD* = 1.60, *d* = 0.44, *r* = 0.000), where those who had not been in contact with COVID-19 positive cases showing more Self-kindness, with a small effect size.

In Common Humanity, significant between-group differences were found by type of treatment [*F* (5, 404) = 7.46, *p* ≤ 0.001], specifically between None and Psychiatric (*M* = 6.59, *SD* = 1.53 vs. *M* = 5.23, *SD* = 1.43, *d* = 0.89, *r* = 0.003), between None and Psychological (*M* = 6.59, *SD* = 1.53 vs. *M* = 5.42, *SD* = 1.98, *d* = 0.74, *r* = 0.002), between Medical and Psychiatric (*M* = 6.44, *SD* = 1.53 vs. *M* = 5.23, *SD* = 1.43, *d* = 0.70, *r* = 0.006), and between Medical and Psychological (*M* = 6.44, *SD* = 1.53 vs. *M* = 5.42, *SD* = 1.98, *d* = 0.79, *r* = 0.007). The results show that the scores in Common Humanity were higher for those with no treatment, with a moderate effect size, and large between None and Psychiatric. The analysis by quality of cohabitation also showed significant between-group differences [*F* (3, 402) = 8.37, *p* ≤ 0.001], particularly between Very Good and Poor (*M* = 6.78, *SD* = 1.64 vs. *M* = 5.41, *SD* = 1.71, *d* = 0.82, *r* = 0.005) and between Good and Poor (*M* = 6.50, *SD* = 1.50 vs. *M* = 5.41, *SD* = 1.71, *d* = 0.70, *r* = 0.002). From the comparison between groups, it may be seen that the scores on Common Humanity were higher in those groups where cohabitation was better, with a large effect size. Finally, statistically significant between-group differences were also found between groups by type of meditation [*F* (4, 396) = 6.10, *p* ≤ 0.001], especially between None and Mindfulness (*M* = 6.23, *SD* = 1.54 vs. *M* = 7.02, *SD* = 1.64, *d* = 0.50, *r* = 0.001) and between None and Transcendental (*M* = 6.23, *SD* = 1.54 vs. *M* = 8.00, *SD* = 1.58, *d* = 1.14, *r* = 0.004). In both cases, the participants who showed more Common Humanity were those who practiced some type of meditation compared to those who did not, with a moderate effect size between those who did not and those who did practice Mindfulness and a large effect size between those who did not practice and those who practiced Transcendental meditation.

Concerning the last subscale, Mindfulness, statistically significant differences were observed between age groups [*F* (2, 402) = 10.35, *p* ≤ 0.001], particularly between Young Adult and Adult (*M* = 6.23, *SD* = 1.75 vs. *M* = 6.74, *SD* = 1.61, *d* = 0.30, *r* = 0.000) and between Young Adult and Middle Aged (*M* = 6.23, *SD* = 1.75 vs. *M* = 7.14 *SD* = 1.58, *d* = 0.54, *r* = 0.001). Mindfulness scores were higher in the older age groups, showing small and moderate effect sizes, respectively. Statistically significant between-group differences were also found by treatment type [*F* (3, 401) = 9.91, *p* ≤ 0.001], especially between None and Psychological (*M* = 6.88, *SD* = 1.65 vs. *M* = 5.35, *SD* = 1.56, *d* = 0.93, *r* = 0.003), between Medical and Psychiatric (*M* = 6.60, *SD* = 1.58 vs. *M* = 5.46, *SD* = 1.78, *d* = 0.70, *r* = 0.006), and between Medical and Psychological (*M* = 6.60, *SD* = 1.58 vs. *M* = 5.35, *SD* = 1.56, *d* = 0.79, *r* = 0.007). In general, those participants who were not under any type of treatment had the highest scores in Mindfulness, with a large effect size, and moderate between Medical and Psychological and Medical and Psychiatric. The analyses by type of meditation also showed statistically significant between-group differences [*F* (4, 396) = 4.83, *p* ≤0.001]—in particular, between those who practiced Mindfulness and the group that did not practice any (*M* = 6.52, *SD* = 1.69 vs. *M* = 7.14, *SD* = 1.55, *d* = 0.37, *r* = 0.000), between those who did not practice any and Transcendental (*M* = 6.52, *SD* = 1.69 vs. *M* = 8.62, *SD* = 1.06, *d* = 1.24, *r* = 0.005), between Mindfulness and Transcendental (*M* = 7.14, *SD* = 1.55 vs. *M* = 8.62, *SD* = 1.06, *d* = 0.97, *r* = 0.012), between Zen and Transcendental (*M* = 6.88, *SD* = 2.23 vs. *M* = 8.62, *SD* = 1.06, *d* = 0.97, *r* = 0.054), and between Vipassana and Transcendental (*M* = 6.33, *SD* = 0.60 vs. *M* = 8.62, *SD* = 1.06, *d* = 2.54, *r* = 0.032). In general, in those groups that practiced meditation, the score on the Mindfulness scale was higher than in those who did not practice any, with a large effect size, except between None and Mindfulness, which was small. Lastly, by type of occupation, statistically significant between-group differences [*F* (6, 398) = 2.96, *p* < 0.05] were also found between Unemployed and Housewife/Husband (*M* = 5.80, *SD* = 1.71 vs. *M* = 7.39, *SD* = 1.90, *d* = 0.90, *r* = 0.014), Unemployed and Student (*M* = 5.80, *SD* = 1.71 vs. *M* = 6.62, *SD* = 1.59, *d* = 0.49, *r* = 0.003), Unemployed and Employed (*M* = 5.80, *SD* = 1.71 vs. *M* = 6.81, *SD* = 1.80, *d* = 0.83, *r* = 0.012), and Unemployed and Government Employee (*M* = 5.80, *SD* = 1.71 vs. *M* = 6.86, *SD* = 1.62, *d* = 0.33, *r* = 0.001). Unemployed scored lower in Mindfulness than the rest of occupations, especially Housewife/Husband, with a larger difference and large effect size in all the comparisons, except Unemployed and Student and Unemployed and Government Employee, where it was small.

The rest of the sociodemographic variables with their mean and standard deviation are shown in Table 2.

### 3.3. Relationships between the Variables Mentioned (Anxiety, Depression, Stress, Self-Compassion) with the Selected Sociodemographic Variables (Ordinal)

After the correlation analysis, older age was observed to be related to higher frequency in practicing meditation, better cohabitation, more Common Humanity, and more Mindfulness. On the contrary, less Stress, Anxiety, and Depression were related with older age. Higher education was significantly related to more frequent practice of Meditation and an increase in the variables in the SCS scale. On the contrary, greater Anxiety, Intrusion, and Avoidance were related to a lower education. More frequent Meditation practice was significantly related to less Depression and an increase in SCS scale variables. Better cohabitation quality was significantly related to higher scores on SCS variables and a lower score on the rest of the scales. Finally, as expected, the psychological scales were significantly correlated with each other (see Table 3).

## 4. Discussion

After analyzing the survey results with regard to the objectives originally set, the participants showed mild depression and anxiety, not reporting symptoms of stress. However, they did report severe symptomatology on the IES scale as in previous studies, which related high scores in posttraumatic stress symptoms or avoidance behaviors after 10 days of quarantine [8]. This difference may be, in part, because the IES scale evaluates the psychological impact of an event after the fact and the participants could have understood that the event referred to COVID-19, but not on the DASS-21 scale.

Another of the objectives of the study proposed Self-compassion and Mindfulness as protective factors against the impact of confinement. It is worth mentioning that the participant scores on the various Self-Compassion Scale subscales (Mindfulness, Self-Kindness, and Common Humanity) were negatively related in both the IES and the DASS-21, suggesting that self-compassion might be an important protective factor, fostering emotional resilience [24]. The results show that higher scores of Self-kindness, Common Humanity, and Mindfulness were related to not being under treatment and in turn, higher scores of Self-kindness and Common Humanity were related to higher cohabitation quality. There was no significant relationship of mindfulness practice with lower scores on the IES or DASS-21 scales, but there was an increase in the Self-Compassion scale, as was to be expected, given the relationship of mindfulness with Self-compassion [25].

The sociodemographic variables that had the strongest relationship with psychological impact were being under treatment or not, age, and quality of cohabitation during the quarantine, indicating that not being under any treatment implied lower scores of Intrusion and Avoidance. Being young was related to higher scores of stress and depression, in agreement with recent studies on the psychological impact, relating age and being a student with higher vulnerability and psychological impact from confinement [14,26]. Furthermore, an improvement in quality of cohabitation was related to a lower Total IES score. On the contrary, worse cohabitation is related to greater Intrusion and Avoidance. These results would suggest that young people under some type of treatment and who report that the quality of their cohabitation is not good, would experience a negative psychological impact of confinement. Specifically, young people show more posttraumatic stress symptomatology, so it would be of interest for authorities to develop psychological interventions, providing a support network during quarantine as previous studies have related an increase in PTSS after similar events, such as Middle East Respiratory Syndrome [27]. It was also observed that cohabitation with a person who was COVID-19 positive implied more intrusive thoughts and avoidance, as well as a higher score on the Total IES, which would be related to experiencing more fear of becoming infected with the virus and in turn, infecting others [10,28,29]. Being Retired was also related to more Intrusion and a higher total score on the IES scale and on the contrary, being a Student was related to less Intrusion and a lower score on the Total IES scale.

This study has some limitations, especially due to the limited resources available and the urgency in its implementation during the state of emergency. We cannot establish any causal relationships between the variables studied. The sample was mostly comprised of women and only an adult population, so it is not a true representation of the entire population. Future studies should have a larger and more homogeneous sample and use a more complex sampling strategy or perform a prospective study that would enable a more specific result to support the need for health initiatives focused on improving the mental health of the participants, promoting attitudes or behaviors for coping with confinement.

In spite of these limitations, the results of this study provide important information on the psychological and social impact of the first three weeks of a COVID-19 quarantine period, including that self-compassion might be an important protective factor, and may be used as a reference in future epidemics or outbreaks of the virus that provoke similar short or long-term situations to generate hypotheses for improvement of prevention and intervention. The results could also provide a baseline for evaluating the psychological and social consequences during the rest of the COVID-19 epidemic, which was still underway at the time of writing.

## 5. Conclusions

During this first stage of confinement due to the COVID-19 outbreak, the participants reported mild anxiety and depression symptomatology, as well as severe posttraumatic stress symptomatology. The quality of cohabitation was shown to be a key variable in the psychological impact of the participants, since its poorer quality was related to higher scores of intrusion and avoidance, which were lower the better the quality of cohabitation was. Being young and being under treatment were also related to a greater psychological impact. Finally, self-compassion was related to better cohabitation during confinement. The findings shown in this study could be used for psychological interventions to improve mental health and coping with confinement during the COVID-19 epidemic.

## Figures and Tables

**Table 1:** Participant characteristics.

Variables	Total	Women	Men
Age group, *n* (%)			
*18–35*	145 (35.2)	122 (29.6)	22 (5.3)
*36–45*	134 (32.5)	114 (27.7)	20 (4.9)
*≥46*	133 (32.3)	112 (27.2)	19 (4.6)
Nationality, *n* (%)			
*Spain*	375 (91)	317 (76.9)	55 (13.3)
*Mexico*	12 (2.9)	10 (2.4)	2 (0.5)
*Peru*	7 (1.7)	7 (1.7)	0 (0)
*Argentina*	3 (0.7)	3 (0.7)	0 (0)
*Ecuador*	1 (0.2)	1 (0.2)	0 (0)
*Paraguay*	2 (0.2)	2 (0.5)	0 (0)
*Rumania*	3 (0.2)	2 (0.5)	1 (0.2)
*Italy*	2 (0.2)	1 (0.2)	1 (0.2)
*UK*	1 (0.2)	1 (0.2)	0 (0)
*Russia*	1 (0.2)	1 (0.2)	0 (0)
*Colombia*	2 (0.2)	1 (0.2)	1 (0.2)
*Lithuania*	1 (0.2)	0 (0)	1 (0.2)
*Brazil*	1 (0.2)	1 (0.2)	0 (0)
*Cuba*	1 (0.2)	1 (0.2)	0 (0)
Marital status, *n* (%)			
*Married*	149 (63.7)	124 (30.2)	23 (5.6)
*Single*	101 (24.6)	86 (20.9)	15 (3.6)
*In a relationship*	60 (14.6)	55 (13.4)	5 (1.2
*Living together*	60 (14.6)	48 (11.7)	12 (2.9)
*Separated*	38 (9.2)	31 (7.5)	6 (1.5)
*Widowed*	3 (0.7)	3 (0.7)	0 (0)
Occupation, *n* (%)			
*Employee*	219 (53.2)	179 (43.4)	37 (9)
*Government employee*	75 (18.2)	66 (16)	9 (2.2)
*Unemployed*	45 (10.9)	39 (9.5)	6 (1.5)
*Student*	31 (7.5)	27 (6.6)	4 (1)
*Retired*	17 (4.1)	15 (3.6)	2 (0.5)
*Housewife*	14 (3.4)	13 (3.2)	1 (0.2)
*Other*	11 (2.7)	9 (2.2)	2 (0.5)
Employment status ^1^, *n* (%)			
*No*	304 (73.8)	255 (61.9)	47 (11.4)
*ERTE/ERE*	51 (12.4)	40 (9.7)	11 (2.7)
*Forced vacation*	21 (5.1)	18 (4.4)	3 (0.7)
*Fired*	18 (4.4)	18 (4.4)	0 (0)
*Other*	18 (4.4)	17 (4.1)	0 (0)
Education, *n* (%)			
*University*	173 (42)	145 (35.2)	26 (6.3)
*Postgraduate*	112 (27.2)	96 (23.3)	16 (3.9)
*Professional training*	64 (15.5)	55 (13.3)	8 (1.9)
*High school*	31 (7.5)	22 (5.3)	9 (2.2)
*Middle school*	22 (5.3)	21 (5.1)	1 (0.2)
*Primary*	10 (2.4)	9 (2.2)	1 (0.2)
Current residence, *n* (%)			
*Family*	233 (56.6)	205 (49.8)	26 (6.3)
*With partner*	113 (27.4)	88 (21.4)	24 (5.8)
*Alone*	52 (12.6)	42 (10.2)	10 (2.4)
*Shared housing*	13 (3.2)	12 (2.9)	1 (0.2)
*Other*	1 (0.2)	1 (0.2)	0 (0)
Current treatment ^2^, *n* (%)			
*None*	304 (73.8)	254 (61.7)	48 (11.7)
*Medical*	67 (16.3)	57 (13.8)	9 (2.2)
*Psychological*	28 (6.8)	25 (6.1)	3 (0.7)
*Psychiatric*	13 (3.2)	12 (2.9)	1 (0.2)
Practice Meditation, *n* (%)			
*No*	284 (68.9)	239 (58)	44 (10.7)
*Yes*	128 (31.1)	109 (26.5)	17 (4.1)
Type of practice, *n* (%)			
*None*	310 (76.7)	259 (64.1)	50 (12.4)
*Mindfulness*	71 (17.6)	64 (15.8)	7 (1.7)
*Zen*	9 (2.2)	7 (1.7)	1 (0.2)
*Transcendental*	8 (2)	6 (1.5)	1 (0.2)
*Vipassana*	6 (1.5)	5 (1.2)	1 (0.2)
COVID-19 diagnosis ^3^, *n* (%)			
*No*	404 (98.8)	342 (83.6)	59 (14.6)
*Yes*	5 (1.2)	3 (0.7)	2 (0.5)
Quality of cohabitation ^4^, *n* (%)			
*Good*	260 (63.6)	219 (53.5)	38 (9.3)
*Very good*	102 (24.9)	83 (20.3)	19 (4.6)
*Poor*	43 (10.5)	39 (9.5)	4 (1)
*Very poor*	4 (1)	4 (1)	0 (0)
Religious, *n* (%)			
*No*	227 (55.1)	191 (46.4)	35 (8.5)
*Yes, but not practicing*	146 (35.4)	128 (31.1)	17 (4.1)
*Yes, practicing*	39 (9.5)	29 (7)	9 (2.2)

^1^ Change in employment due to COVID-19; ^2^ type of treatment now under; ^3^ Diagnosed with COVID-19 by a healthcare professional; ^4^ Opinion of cohabitation during quarantine.

**Table 2:** Means and standard deviations of the Depression, Anxiety and Stress Scale-21 (DASS-21), Self-Compassion Scale (SCS), and Impact of Events Scale (IES) for sociodemographic variables.

Variables	Stress *M (SD*)	Anxiety *M (SD*)	Depression *M (SD*)	Self-Kindness *M (SD*)	Common Humanity *M (SD)*	Mindfulness *M (SD)*	Intrusion *M* (*SD*)	Avoidance *M (SD)*
Age group (*n* = 412)								
*<35* (*n* = 145)	7.73 (5.20)	4.48 (4.80)	5.21 (4.64)	6.24 (1.70)	6.23 (1.69)	6.23 (1.75)	12.49 (6.78)	13.60 (7.44)
*36–45* (*n* = 134)	6.44 (4.83)	3.88 (4.41)	4.48 (4.42)	6.55 (1.64)	6.42 (1.49)	6.74 (1.61)	12.59 (7.68)	13.16 (7.93)
*>46* (*n* = 133)	5.76 (5.54)	3.22 (4.20)	3.79 (4.47)	6.62 (1.63)	6.70 (1.58)	7.14 (1.58)	12.77 (7.75)	14.22 (7.52)
Education (*n* = 412)								
*Primary* (*n* = 10)	6.60 (5.98)	5.30 (5.43)	4.80 (4.61)	5.95 (1.14)	6.15 (0.74)	6.10 (1.39)	17.22 (6.55)	16.55 (8.48)
*Secondary* (*n* = 22)	8.18 (5.72)	6.77 (5.68)	6.54 (5.20)	5.61 (1.63)	5.61 (1.85)	5.80 (2.10)	16.18 (7.81)	14.95 (6.80)
*High School* (*n* = 31)	6.67 (5.14)	3.80 (3.81)	5.35 (4.17)	6.25 (1.47)	6.27 (1.72)	6.45 (1.60)	12.10 (6.91)	15.32 (7.97)
*Professional Training* (*n* = 64)	6.60 (5.62)	4.45 (5.22)	4.96 (5.32)	6.23 (1.68)	6.24 (1.35)	6.69 (1.87)	12.81 (7.61)	13.58 (8.33)
*University* (*n* = 173)	6.35 (5.32)	3.68 (4.38)	4.08 (4.32)	6.48 (1.58)	6.43 (1.49)	6.77 (1.53)	13.06 (7.40)	13.99 (7.63)
*Postgraduate* (*n* = 112)	6.92 (4.83)	3.20 (3.87)	4.27 (4.28)	6.84 (1.89)	6.83 (1.79)	6.86 (1.76)	10.88 (6.98)	12.26 (7.06)
Type of treatment (*n* = 430)								
*None* (*n* = 352)	6.06 (4.84)	3.24 (3.98)	3.87 (4.00)	6.70 (1.56)	6.59 (1.53)	6.88 (1.65)	11.73 (7.08)	12.88 (7.48)
*Medical* (*n* = 78)	7.52 (5.83)	5.26 (5.09)	5.49 (4.91)	5.91 (1.63)	6.44 (1.53)	6.60 (1.58)	14.89 (7.84)	16.01 (8.04)
*Psychiatric* (*n* = 352)	10.76 (6.71)	7.15 (6.75)	7.76 (7.47)	5.96 (2.26)	5.23 (1.43)	5.46 (1.78)	15.53 (6.62)	15.07 (6.56)
*Psychological* (*n* = 352)	9.42 (5.59)	6.03 (5.37)	7.64 (5.34)	5.46 (1.87)	5.42 (1.98)	5.35 (1.56)	15.35 (8.02)	16.00 (7.18)
Cohabitation quality (*n* = 430)								
*Very good* (*n* = 352)	4.27 (4.55)	2.28 (3.60)	2.43 (3.30)	8.99 (10.36)	6.91 (1.64)	7.30 (1.59)	9.67 (7.33)	10.93 (7.33)
*Good* (*n* = 78)	6.60 (4.72)	3.73 (4.13)	4.35 (3.93)	14.69 (11.60)	6.51 (1.59)	6.71 (1.56)	12.84 (6.99)	14.07 (7.50)
*Poor* (*n* = 352)	11.88 (5.08)	8.02 (5.20)	9.79 (5.80)	29.69 (14.69)	5.18 (1.58)	5.26 (1.83)	17.57 (6.91)	17.21 (7.29)
*Very poor* (*n* = 352)	15.75 (7.93)	11.00 (9.34)	11.75 (5.12)	38.50 (19.48)	5.87 (2.39)	4.62 (0.47)	18.25 (7.88)	17.00 (7.07)
Type of practice (*n* = 430)								
*None* (*n* = 352)	6.72 (5.27)	4.00 (4.72)	4.89 (4.74)	6.28 (1.55)	6.23 (1.54)	6.52 (1.69)	12.70 (7.70)	13.35 (7.79)
*Mindfulness* (*n* = 78)	6.05 (4.68)	3.12 (3.01)	3.33 (3.69)	7.09 (1.92)	7.02 (1.64)	7.14 (1.56)	11.64 (6.52)	13.94 (7.58)
*Zen* (*n* = 352)	10.22 (8.25)	7.88 (7.09)	4.55 (4.82)	6.94 (1.70)	6.83 (1.75)	6.88 (2.23)	16.88 (6.56)	17.55 (5.76)
*Vipassana* (*n* = 352)	10.88 (4.26)	6.33 (4.27)	4.66 (4.36)	5.5 (1.30)	6.83 (1.03)	6.33 (0.60)	11.83 (8.37)	17.60 (5.54)
*Transcendental (n = 321)*	4.37 (3.50)	2.25 (1.75)	2.00 (1.60)	7.62 (2.18)	8.00 (1.58)	8.62 (1.06)	11.75 (2.96)	12.62 (3.81)

**Table 3:** Correlation coefficients between sociodemographic variables and the subscales evaluated.

−	Age	Education	Mindfulness Frequency	Cohabitation Quality	Stress	Anxiety	Depression	Intrusion	Avoidance
Education	−0.73								
Mindfulness frequency	0.129 **	0.172 **							
Cohabitation quality ^1^	0.206 **	0.066	0.095						
Stress	−0.190 **	0.010	−0.007	−0.385 **					
Anxiety	−0.145 **	−0.121 *	−0.018	−0.346 **	0.771 **				
Depression	−0.175 **	−0.087	−0.127 **	−0.419 **	0.786 **	0.697 **			
Self-kindness	0.052	0.193 **	0.181 **	0.237 **	−0.318 **	−0.363 **	−0.422 **		
Common humanity	0.119 *	0.165 **	0.196 **	0.190 **	−0.306 **	−0.266 **	−0.390 **		
Mindfulness	0.194 **	0.116 *	0.157 **	0.297 **	−0.465 **	−0.468 **	−0.552 **		
Intrusion	0.040	−0.145 **	−0.028	−0.297 **	0.626 **	0.586 **	0.566 **		
Avoidance	0.012	−0.117 *	0.78	−0.242 **	0.476 **	0.465 **	0.458 **	0.711 **	

^1^ Cohabitation quality during confinement; * *p* < 0.05, ** *p* < 0.001.

## References

[1] Hawryluck L., Gold W.L., Robinson S., Pogorski S., Galea S., Styra R. (2004). SARS control and psychological effects of quarantine, Toronto, Canada. Emerg. Infect. Dis..

[2] Mahase E. (2020). China coronavirus: WHO declares international emergency as death toll exceeds 200. BMJ.

[3] WHO Considerations for Quarantine of Individuals in the Context of Containment for Coronavirus Disease (COVID-19). https://www.who.int/publications-detail/considerations-for-quarantine-of-individuals-in-the-context-of-containment-for-coronavirus-disease-(covid-19).

[4] WHO Coronavirus Disease (COVID-2019) Situation Reports. Report 44. https://www.who.int/docs/default-source/coronaviruse/situation-reports/20200304-sitrep-44-covid-19.pdf?sfvrsn=783b4c9d_2.

[5] Cruz M.P., Santos E., Cervantes M.V., Juárez M.L. (2020). COVID-19, una emergencia de salud pública mundial. Rev. Clín. Esp..

[6] Ryu S., Chun B.C. (2020). Korean Society of Epidemiology 2019-nCoV Task Force Team An interim review of the epidemiological characteristics of 2019 novel coronavirus. Epidemiol. Health.

[7] Liu Y., Gayle A.A., Wilder-Smith A., Rocklov J. (2020). The reproductive number of COVID-19 is higher compared to SARS coronavirus. J. Travel Med..

[8] Carlo D.T.D., Montemurro N., Petrella G., Siciliano G., Ceravolo R., Perrini P. (2020). Exploring the clinical association between neurological symptoms and COVID-19 pandemic outbreak: A systematic review of current literature. J. Neurol..

[9] Rogers J.P., Chesney E., Oliver D., Pollak T.A., McGuire P., Fusar-Poli P., Zandi M.S., Lewis G., David A.S. (2020). Psychiatric and neuropsychiatric presentations associated with severe coronavirus infections: A systematic review and meta-analysis with comparison to the COVID-19 pandemic. Lancet Psychiatry.

[10] Brooks S.K., Webster R.K., Smith L.E., Woodland L., Wessely S., Greenberg N., Rubin G.J. (2020). The psychological impact of quarantine and how to reduce it: Rapid review of the evidence. Lancet.

[11] Zhang Y., Ma Z.F. (2020). Impact of the COVID-19 pandemic on mental health and quality of life among local residents in Liaoning Province, China: A cross-sectional study. Int. J. Environ. Res. Public Health.

[12] Koolhaas J.M., Bartolomucci A., Buwalda B., Boer S.D., Flügge G., Korte S.M., Meerlo P., Murison R., Olivier B., Palanza P. (2011). Stress revisited: A critical evaluation of the stress concept. Neurosci. Biobehav. Rev..

[13] Liu N., Zhang F., Wei C., Jia Y., Shang Z., Sun L., Wu L., Sun Z., Zhou Y., Wang Y. (2020). Prevalence and predictors of PTSS during COVID-19 outbreak in China hardest-hit areas: Gender differences matter. Psychiatry Res..

[14] Cao W., Fang Z., Hou G., Han M., Xu X., Dong J., Zheng J. (2020). The psychological impact of the COVID-19 epidemic on college students in China. Psychiatry Res..

[15] Boyd J.E., Lanius R.A., McKinnon M.C. (2018). Mindfulness-based treatments for posttraumatic stress disorder: A review of the treatment literature and neurobiological evidence. J. Psychiatry Neurosci..

[16] Davis L.L., Whetsell C., Hamner M.B., Carmody J., Rothbaum B.O., Allen R.S., Bartolucci A., Southwick S.M., Bremner J.D. (2019). A multisite randomized controlled trial of mindfulness-based stress reduction in the treatment of posttraumatic stress disorder. Psychiatr. Res. Clin. Pract..

[17] Winders S., Murphy O., Looney K., O’Reilly G. (2020). Self-compassion, trauma, and posttraumatic stress disorder: A systematic review. Clin. Psychol. Psychother..

[18] Lovibond P.F., Lovibond S. (1995). The structure of negative emotional states: Comparison of the Depression Anxiety Stress Scales (DASS) with the Beck Depression and Anxiety Inventories. Behav. Res. Ther..

[19] Daza P., Novy D.M., Stanley M.A., Averill P. (2002). The depression anxiety stress scale-21: Spanish translation and validation with a Hispanic Sample. J. Psychopathol. Behav. Assess..

[20] Horowitz M., Wilner N., Alvarez W. (1979). Impact of event scale: A measure of subjective stress. Psychosom. Med..

[21] Alonso J., Prieto L., Antó J.M. (1995). La versión española del “SF-36 Health Survey” (Cuestionario de Salud SF-36): Un instrumento para la medida de los resultados clínicos. Med. Clin..

[22] Neff K.D. (2003). The development and validation of a scale to measure self-compassion. Self Identity.

[23] García-Campayo J., Navarro-Gil M., Andrés E., Montero-Marin J., López-Artal L., DeMarzo M. (2014). Validation of the Spanish versions of the long (26 items) and short (12 items) forms of the Self-Compassion Scale (SCS). Health Qual. Life Outcomes.

[24] Neff K. (2003). Self-Compassion: An alternative conceptualization of a healthy attitude toward oneself. Self Identity.

[25] Wang C., Pan R., Wan X., Tan Y., Xu L., Ho C.S.H., Ho R.C. (2020). Immediate psychological responses and associated factors during the initial stage of the 2019 Coronavirus Disease (COVID-19) epidemic among the general population in China. Int. J. Environ. Res. Public Health.

[26] Bacas D.C., Martí A.J.C.I., Cantó P.R. (2015). Mindfulness estado, habilidades mindfulness y auto-compasión en el aprendizaje de mindfulness: Un estudio piloto. Àgora Salut.

[27] Sandin B., Valiente R.M., García-Escalera J., Chorot P. (2020). Impacto psicológico de la pandemia de COVID-19: Efectos negativos y positivos en población española asociados al periodo de confinamiento nacional. J. Psychopathol. Clin. Psychol..

[28] Jeong H., Yim H.-W., Song Y.-J., Ki M., Min J.-A., Cho J., Chae J.-H. (2016). Mental health status of people isolated due to Middle East Respiratory Syndrome. Epidemiol. Health.

[29] Montemurro N. (2020). The emotional impact of COVID-19: From medical staff to common people. Brain Behav. Immun..

